# The Organoid Era Permits the Development of New Applications to Study Glioblastoma

**DOI:** 10.3390/cancers12113303

**Published:** 2020-11-09

**Authors:** Francesco Andreatta, Giulia Beccaceci, Nicolò Fortuna, Martina Celotti, Dario De Felice, Marco Lorenzoni, Veronica Foletto, Sacha Genovesi, Josep Rubert, Alessandro Alaimo

**Affiliations:** 1 Department of Cellular, Computational and Integrative Biology (CIBIO), University of Trento, Via Sommarive 9, 38123 Trento, Italy; francesco.andreatt-1@studenti.unitn.it (F.A.); giulia.beccaceci@studenti.unitn.it (G.B.); nicolo.fortuna@studenti.unitn.it (N.F.); mcelotti@bccrc.ca (M.C.); dario.defelice@unitn.it (D.D.F.); marco.lorenzoni90@gmail.com (M.L.); veronica.foletto@unitn.it (V.F.); sacha.genovesi@unitn.it (S.G.); josep.rubert@unitn.it (J.R.); 2 Interdisciplinary Research Structure of Biotechnology and Biomedicine, Department of Biochemistry and Molecular Biology, Universitat de Valencia, 46100 Burjassot, Spain

**Keywords:** glioblastoma, organoids, tumoroids, preclinical cancer models, stem cells, translational research, precision medicine

## Abstract

**Simple Summary:**

Glioblastoma is the most lethal primary adult brain tumor. The great number of mutations involved and the aggressiveness of glioblastoma render this type of cancer especially difficult to investigate. To address this problem, cerebral organoids have emerged as promising tools to investigate brain biology and to recapitulates the major steps involved in glioblastoma tumorigenesis. This review focuses on methods of cerebral organoid development, describes the protocols used for inducing glioblastoma, the approach used to derive glioblastoma organoids directly from patients’ biopsies and discusses their limitations and potential future direction.

**Abstract:**

Glioblastoma (GB) is the most frequent and aggressive type of glioma. The lack of reliable GB models, together with its considerable clinical heterogeneity, has impaired a comprehensive investigation of the mechanisms that lead to tumorigenesis, cancer progression, and response to treatments. Recently, 3D cultures have opened the possibility to overcome these challenges and cerebral organoids are emerging as a leading-edge tool in GB research. The opportunity to easily engineer brain organoids via gene editing and to perform co-cultures with patient-derived tumor spheroids has enabled the analysis of cancer development in a context that better mimics brain tissue architecture. Moreover, the establishment of biobanks from GB patient-derived organoids represents a crucial starting point to improve precision medicine therapies. This review exemplifies relevant aspects of 3D models of glioblastoma, with a specific focus on organoids and their involvement in basic and translational research.

## 1. Introduction

Glioblastoma (GB), also known as glioblastoma multiforme, is the most aggressive type of brain cancer. It accounts for 14.6% of all primary brain and other central nervous system (CNS) tumors, 48.3% of primary malignant brain tumors, and 57.3% of all gliomas in adults [1]. GB is a form of glioma, a cluster of cancers that has long been thought to arise from glial cells of the CNS. However, recently, strong evidence has disclosed that GB arises from neural stem cells within the subventricular zone of the brain rather than mature glia [2].

Two broad classes of infiltrative gliomas are histologically identified according to normal glial populations: astrocytomas, that resemble astrocytes, and oligodendrogliomas, which have oligodendrocytes as their normal morphological counterparts [3]. Gliomas are further graded and categorized according to the World Health Organization (WHO) guidelines, which are based on a combination of histologic and molecular features [4]. The most advanced astrocytomas (grade IV) are classified as glioblastoma. Besides atypical glial cells, the essential diagnostic features of GB are brisk mitotic activity, evidence of microvascular proliferation (MVP), and significant necrosis. MVP typically appears as glomeruloid tufts of multilayered endothelial cells that are mitotically active along with smooth muscle cells or pericytes [5]. Because of extensive neo-angiogenesis, the vasculature is highly abnormal with leaky and hyper dilated vessels. Necrosis is a fundamental feature of GB and the strongest predictor of aggressiveness [6,7,8].

Despite several clinical trials performed in the last 15 years, the therapeutic options for primary glioblastoma have remained limited: the standard-of-care therapy consists in maximal surgical resection of the aberrant tissue in combination with chemotherapy, based on the alkylating agent temozolomide, and radiation treatment [9]. Median survival has remained mostly unchanged for 30 years [10] and this treatment regimen extends it up to 15 months after the initial diagnosis [9]. Nevertheless these aggressive treatments, recurrence is almost inevitable and no standard cure has been outlined [11]. Possible approaches for recurrent glioblastoma include re-resection, treatment with the anti-angiogenesis agent bevacizumab and experimental therapies in the context of clinical trials. Unfortunately, none of these approaches showed significantly increased survival rate. Indeed, for recurrent GB patients, the six-month progression-free survival is ~15% and the overall survival is less than six months [12,13,14].

Two aspects that pose a significant challenge in the treatment of GB are the extensive intra- and intertumoral heterogeneity [15,16] and the highly invasive nature of these tumors [17,18,19,20]. The ability of glioblastoma cells to infiltrate healthy brain tissue depends on complex interactions between tumor cells and the surrounding microenvironment, consisting of microglia, bone marrow-derived macrophages, astrocytes, oligodendrocytes, neurons, glial and neuronal progenitors, pericytes, endothelial cells, and extracellular matrix (ECM) [21]. Both experimental and histological evidences indicate that GB cells migration is accompanied by the expression of stem cell markers, which can be predictive of patient outcomes [22,23,24]. Indeed, multiple studies have suggested the presence of a small sub-population of tumor-initiating and tumor-propagating neural stem-like cells called cancer stem cells (CSCs) or, specifically for GB, glioma stem cells (GSCs) [25,26,27]. GSCs reside both in perivascular niches, where the close proximity to the vasculature provides nutrients and oxygen [28], and in hypoxic regions distal to the blood vessels [29,30,31]. Interestingly, GSCs were uncovered to be resistant to conventional therapies through multiple mechanisms, including increased DNA repair [32]. Overall, (i) the simultaneous presence of different stem, progenitor and differentiated cells, (ii) the high degree of intra- and inter-tumoral heterogeneity and (iii) the complex network between tumor cells and their surrounding microenvironment, render in vitro modeling of GB particularly challenging.

In this intricate panorama, the establishment of refined model systems of GB is imperative. Specifically, it is necessary to implement innovative models that closely recapitulate the multitude of phenotypes involved in GB, thus enabling the design of new therapeutic approaches. Several laboratories have directed their efforts to generate organoid models of glioblastoma, which consist, by definition, of 3D structures in which different cell types self-organize to establish appropriate cell-cell contacts and to create a microenvironment [33]. The following chapters describe relevant GB models developed up to now, highlighting how glioblastoma organoids have recently emerged as promising platforms to investigate GB tumor biology.

## 2. Experimental Models to Investigate GB

The vast number of mutations involved, the broad heterogeneity of phenotypic outcomes and the aggressiveness of GB render this type of cancer especially difficult to investigate. Over the last few decades, fundamental preclinical models have been generated to study the mechanisms that lead to GB onset and progression (Table 1).

### 2.1. 2D Models

Historically, cancer cell lines have been models easy to handle in order to study tumor molecular biology and performing drug screening. Many GB immortalized cell lines, including U87, U251 and T98G, have been established in the past years to investigate the mechanisms related to GB biology [34]. However, over the passages in standard serum-containing medium, human GB cell lines present a high amount of genotypic and transcriptomic alterations that often result in little resemblance with the tumor of origin [35,36]. Moreover, when transplanted in nude mice, human GB cell lines often became more homogeneous than the tumor they were derived from [37] and showed limited necrosis and microvascular alterations. Taken together, these features render GB cell lines a flawed model to investigate GB development [13,38].

An emerging field of research has hypothesized that the CSCs model can be applied to GB, thus explaining the high level of heterogeneity and the intrinsic resistance to therapies experienced in clinics. Therefore, GSCs monolayer culturing methods have been established. GSCs are passaged in serum-free medium supplemented with epidermal growth factor (EGF), fibroblast growth factor (FGF2), and the supplements B-27 and N-2 [39,40,41]. GSCs retain phenotypic and genomic features of the original tumor and may be successfully employed to investigate the effects of specific mutations when engineered via gene-editing techniques. However, when cultured in adherent conditions, they are unable to assess the 3D microenvironmental interactions that occur in vivo and are not suitable to investigate the invasive potential of cancer cells on the surrounding healthy tissue [42,43]. Likewise, GSCs have been proved not to be totally trustworthy in the design of new drugs [44].

Finally, when transplanted intracranially in immunocompromised mice, GSCs have been shown to generate vascularized tumors that only partially resemble the histopathological features of human GB. In fact, GSC-derived tumors retain heterogeneity observed in the cellular population of origin and exhibit an invasive potential, while necrosis was not always observed in vivo [39,40].

### 2.2. Preclinical In Vivo Mouse Models

Given the complexity of environmental influences and cell-to-cell interactions in the brain, in vivo preclinical models have been established to investigate the mechanisms that lead to GB development.

#### 2.2.1. Genetically Engineered Mouse Models

Among all the available models, genetically engineered mouse models (GEMMs) have been exploited to study the etiology and molecular basis of GB and the phenotypic effects in a spatial and temporal context. Importantly, GEMMs can be used to assess tumor progression in a microenvironment that resembles the conditions of endogenous cancer onset. Generally, mice do not need to be immunocompromised and retain all cellular players, such as endothelial cells, that are involved both in physiology and in tumorigenesis [45]. However, their applications are limited because GEMMs are an expensive and a time-consuming model [13]. The intrinsic differences between human and rodent cerebral features may lead to misleading interpretations when investigating genetic drivers and treatment responses [46]. Finally, GEMMs are not a reliable platform to resemble human tumor heterogeneity.

#### 2.2.2. Mouse Embryonic Brains

Establishing a GEMM model is a time-consuming process. To overcome this issue, other strategies have been applied to generate GB models in mice. Specifically, one approach that has been successfully employed consists in genetic modification of the developing mouse embryonic brain directly in utero via electroporation, exploiting CRISPR/Cas9 mediated gene editing [47,48]. This model may represent a faster method that allows for the investigation of more putative GB-related genes. However, the manipulation of embryos in the uterus remains a crucial technical challenge [49].

### 2.3. In Vitro 3D Models

#### 2.3.1. Spheroids

Spheroids are currently the most used 3D cultures for GB. They mirror a realistic in vitro scenario of tumor growth and invasion [50]. Furthermore, they are widely adopted for high-throughput drug screening since they are easy to handle and engineer [50,51,52]. Spheroids are usually derived from GB cell lines growing as spheres in a 3D matrix or in suspension. Yet, they often present a discrepancy in gene expression compared to the primary tissue and, in general, they cannot capture the molecular and histological heterogeneity of patients [36,53].

To partially overcome the problem, tumorspheres obtained from tumor-derived stem cells have also been established. These cells can differentiate in neural cells (neurons, astrocytes or oligodendrocytes) with a similar proportion to the parental tumor [54]. Importantly, tumorspheres present an hypoxic core and a highly proliferating periphery [55,56]. Despite the relevant insights that this model has probed on stemness properties and drug resistance [55,57], tumorspheres are highly unstable and, after few passages, a clonal expansion of a specific cell population can be observed [58]. Moreover, cells growing in Matrigel™ and EGF/FGF2 containing media may be influenced by these exogenous factors.

Human GB spheroids have also been employed in xenotransplantations to analyze the mechanisms of tumor progression in vivo. When implanted in nude mice, GB spheroids can closely recreate the hierarchical cell organization and heterogeneity of the parental tumor [23]. Thus, they may allow the investigation of tumor biology when cancer is still at an early stage, before the insurgence of evident symptoms [59].

#### 2.3.2. Organoids

**Figure 1:**
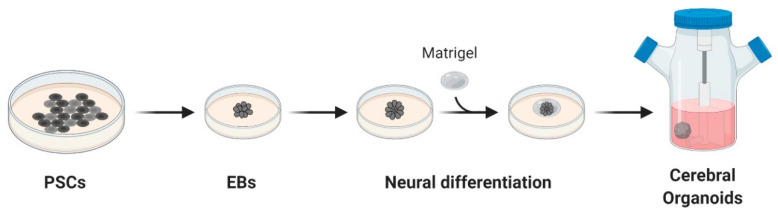
Schematic overview of cerebral organoids’ derivation. Cerebral organoids were obtained starting from pluripotent stem cells (PSCs) cultured as embryoid bodies (EBs) and successively differentiated to generate neuroectoderm. These 3D cultures, embedded in droplets of Matrigel, were then moved to a spinning-bioreactor containing differentiation media. Adapted from reference [61].

Organoids are 3D structures usually developed from patient-derived stem cells embedded in a matrix, commonly Matrigel™, and cultured with a cocktail of growth factors. These cells proliferate and differentiate in a few days, self-organizing in an organotypic structure [60]. In 2013, Lancaster and colleagues generated a robust protocol for the derivation of cerebral organoids. Starting from induced pluripotent stem cells (iPSCs), cultured as embryoid bodies, they induced differentiation towards the neuroectoderm and embedded the cells in Matrigel^TM^ droplets. These droplets were then cultured in a differentiation media containing EGF/FGF2 and moved to a spinning bioreactor (Figure 1) [61].

Notably, the organoid structural organization recapitulates the early stages of a developing human brain [62,63]. Neurons maturate with a pyramidal identity with modest spatial separation and, more importantly, they display a high outer radial glia population, a stem cells zone which is limited in rodents. Still, it is essential in human brain development [64]. For these reasons, cerebral organoids have been widely used to recapitulate developmental phases of the neural tissue and to model neurodevelopmental disorders *in vitro* [65,66,67]. Recently, it has been shown that cerebral organoids exhibit good reproducibility, with an organoid-to-organoid variability comparable to that of individual endogenous brains, and deliver consistency in the cell types produced [68].

In the cancer field, organoids are often used to capture the patients’ heterogeneity and, thanks to the many established biobanks, they will probably become a valuable tool for drug screening and precision medicine [60,69]. However, organoids still present significant limitations as they lack stromal components, blood vessels and immune cells, even if some co-cultures have already been established [70,71,72,73]. Moreover, the ECM-like matrix usually used to culture organoids contains extrinsic factors that may influence experimental outcomes, a severe impairment that could be overcome with the definition of a synthetic matrix [74].

Remarkably, human cerebral tumoroids have been employed to perform orthotopic transplantation in immunocompromised mice. A growing body of evidence has observed that GB cerebral tumoroid xenografts show an invasive phenotype, retain stem-like features and display a hypoxia gradient within the tumor infiltrating mass that closely resembles the characteristics observed in clinics [75,76].

In this review, we will describe organoids application in glioblastoma research: cerebral organoids can be genetically modified to study tumor initiation [46,77] or co-cultured with patient derived tumor spheroids to investigate the invasiveness potential [78,79]. Moreover, it is also possible to derive organoids directly from patients’ biopsies for translational studies, better resembling the tumor microenvironment (TME) [75,76].

**Table 1:** Advantages and disadvantages of glioblastoma models.

Model	Advantages	Limitations
Genetically Engineered Mice [80,81,82]	Investigation of phenotypic consequences of GB progression (e.g., tissue invasion)	Lack of clinical validation Difficulties in reproducing human tumor heterogeneity Expensive and time consuming
Mouse embryonic brains [47,48]	Feasibility to investigate immune interactions	Difficulties in assessing clinical relevance Technical issues due to in utero electroporation
Human stem cells [83,84,85]	Possibility to investigate human GB onset Easiness in experimental standardization	Absence of fundamental physiological components (e.g., immune and endothelial cells)
Human cerebral organoids [46,77]	Assessment of human GB development, microenvironmental interactions in a 3D context Possibility to co-culture cancer cells with healthy neuronal cells	Lack of fundamental physiological components (e.g., immune and endothelial cells)

## 3. Genetic Engineering Applied to Cerebral Organoids and Other GB Models

Glioblastoma is a highly heterogeneous type of brain cancer. Several genetic alterations have been described to be involved in the onset of the disease, including the amplification of epidermal growth factor receptor (*EGFR*) gene, mutations in isocitrate dehydrogenase (*IDH*), telomerase reverse transcriptase (*TERT*), phosphatase tensin homologue (*PTEN*), neurofibromatosis type 1 (*NF1*), platelet-derived growth factor receptor alpha (*PDGFRα*), tumor protein p53 (*TP53*), retinoblastoma protein (*RB*), cyclin-dependent kinase inhibitor 2A (*CDKN2A*) and altered promoter methylation of O6-Methylguanine-DNA methyltransferase (*MGMT*) [5,86,87,88].

Although the broad panorama of genetic alterations described, a comprehensive genomic analysis performed within The Cancer Genome Atlas (TCGA) program has established that GB-related genetic lesions can be grouped in three main pathways: *RTK/RAS/PI3K* pathway (88% of cases) and *TP53/RB* tumor suppressive pathways (87% and 78% of cases, respectively) [89].

To investigate these dysfunctions on tumor onset and progression, various experimental models of GB have been established through genetic engineering (Table 1). Among the *in vitro* models, cerebral organoids derived from human tissues present the advantages of mimicking the in vivo structure and the environmental interactions and, compared to mouse-derived models, have a more reliable clinical relevance. The gene-editing of human brain organoids has enabled the study of early phases of tumorigenesis and cancer progression taking into account all these variables.

**Figure 2:**
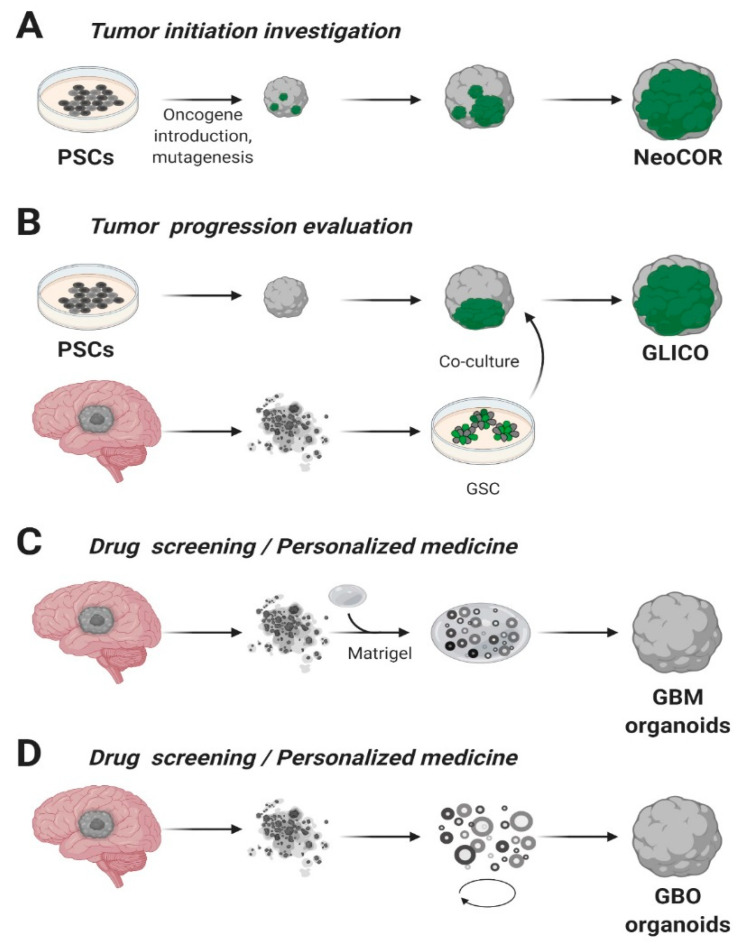
Glioblastoma organoids. (**A**) Cerebral organoids, derived from pluripotent stem cells (PSCs) or embryonic stem (ES) cells, can be genetically engineered introducing tumor-promoting mutations or oncogenes and green fluorescent protein (GFP) to visualize tumor growth. NeoCOR, neoplastic cerebral organoids [46,77]. (**B**) Glioblastoma stem cells (GSCs) marked with GFP have been co-cultured with cerebral organoids to obtain glioma cerebral organoids (GLICO) [77,79]. Patient-derived Glioblastoma (GBM) specimens have been embedded in Matrigel (**C**) [75] or cultured in Matrigel- and serum-free conditions, on a spinning-bioreactor (**D**) [76], to obtain GB organoids. Adapted from reference [43].

A pioneering and ambitious study was carried out by Bian and colleagues, in which they combined Sleeping Beauty transposon-mediated oncogene insertion with CRISPR/Cas9 mutagenesis of tumor suppressor genes [46]. The authors generated an in vitro 3D model called “neoplastic cerebral organoid” (neoCOR), which allowed them to recapitulate some of the most common and clinically relevant combinations of gain or loss of function mutations observed in GB and other brain tumors such as medulloblastoma (Figure 2A). Specifically, they generated three GB models carrying the following mutations: *CDKN2A^−/^CDKN2B^−/^EGFR^OE/^EGFRvIII^OE^* (GBM-1), *NF1^−/^PTEN^−/^TP53^−^* (GBM-2) and *EGFRvIII^OE/^CDKN2A^−/^PTEN^−^* (GBM-3). GB-like organoids displayed a transcriptomic profile comparable to that observed in patients and presented markers typically associated with the GB phenotype in clinics. NeoCORs exhibited several glial markers, such as S100β and GFAP, and they were positive for the proliferative marker Ki67 and other neoplastic markers. Interestingly, GB-like neoCORs xenotransplanted in immunocompromised mice could proliferate, generating neoplastic-like regions characterized by local tissue invasion. At the same time, GB-like organoids were also shown to be suitable for drug screening. The authors tested an EGFR inhibitor, afatinib, currently used in a clinical trial to treat GB (ClinicalTrials.gov, No.: NCT02423525), observing a drastic decrease of tumor cells in two out of three GB-like neoCORs, namely GBM-1 and GBM-3 [46].

Similarly, Ogawa and collaborators established genetically engineered human cerebral organoids exploiting the CRISPR/Cas9 technology to insert a copy of *HRAS^G12V^* by homologous recombination in the *TP53* locus [77]. The authors obtained a small percentage of cells that were genetically modified with mutant *RAS* expression simultaneously with *TP53* tumor suppressor gene disruption and they co-cultured transformed cells with wild type ones. Notably, maintaining organoids in culture for several weeks, they observed that the ratio of modified cells over the total increased overtime, and that engineered cells showed an invasive phenotype. Cancerous cells generated mass projections over the organoid boundary, displaying high levels of Ki67 proliferation marker. The expression profile of these tumoroids was strikingly similar to the mesenchymal subtype of clinical human glioblastoma, presenting high invasive ability both in vitro and in vivo after xenotransplantation in immunocompromised mice [77].

Despite the relatively recent history of this technology, genetic engineering of human cerebral organoids has been proved to allow the generation of *in vitro* models, which combine some of the mutations most frequently observed in clinics, and analyze phenotypes and molecular consequences in a specific genetic context. Considering the difficulties of collecting patient-derived samples, particularly at the early stages of GB, human brain organoids may open new horizons to generate a reliable platform to investigate GB onset and progression, to analyze important GB hallmarks, such as cancer cell invasive capacity, and to perform drug screening [13] (Table 2).

## 4. Co-Cultures of Cerebral Organoids

To mimic the impact of TME in GB and the glioblastoma complexity in vitro, several techniques have been developed. Among them, the co-culture system seems to be the most promising. Specifically, co-cultures provide a good representation of the human in vivo-like tissue model, giving insights on the natural interactions between cell populations [90]. Moreover, the presence of another cell population has been shown to improve the culturing success and cell behavior [90]. Recently, the development of glioma spheres to three-dimensional culture has prompted researchers to couple co-culture with this new technology. More in depth, artificial 3D culture platforms provide an additional dimension for cellular proliferation and interaction compared to 2D models, facilitating the spatial organization of cell morphology and cell-cell or cell-ECM signal transduction [91].

In the GB tumor microenvironment, the crosstalk between neoplastic cells and the surrounding stroma, including microglia, macrophages, astrocytes and neural stem cells, contributes to tumor initiation, progression and metastasis [92]. Microglia and astrocytes constitute most of the non-cancerous cells in glioblastoma, representing approximately 30–50% of the tumor mass [93]. In a healthy brain, they are responsible for maintaining brain homeostasis. However, it has been shown that they stimulate the proliferation and invasion of GB cells in vitro and in vivo, thus, helping tumor cells to create an immunosuppressive environment [94].

Leite and collaborators proved that 3D co-culture of human GB cell lines and microglia supports glioblastoma growth and migration creating a protective environment for GB [95]. They also displayed a new potential role of microglia in glioblastoma: microglia appear to modulate sensitivity to cytotoxic agents conferring drug resistance to the tumor. Similarly, another study hypothesizes that astrocytes in TME behave like microglia by reducing cancer sensitivity to drugs and interactions between astrocytes and GB cells could be associated with increased growth and invasion of the tumor [96]. To confirm this, it was demonstrated that the direct contact between 3D co-culture of astrocytes and GB cells enhances glioblastoma formation [97]. More in detail, the authors of this study suggested that astrocytes may rescue the damaged target cancer cells transferring organelles along tunneling nanotubes. These observations denote an essential role of non-neoplastic cells in the tumor tissue and provide a necessary basis to develop new strategies for glioblastoma treatment.

On the other hand, Da Silva and colleagues adopted the co-culture system to model the invasiveness of GB tumor cells [78]. They highlighted how a co-culture of spheroids derived from GB cells or neural progenitors can infiltrate in early-stage cerebral organoids, resulting in the formation of hybrid organoids that exhibit the phenotype of an invasive tumor. Based on these findings, starting from 3D human embryonic stem cells or patients’ iPSCs, cerebral organoids were obtained and co-cultured with patient-derived GSCs. The injected glioma stem cells had the capability to penetrate in the cerebral organoids, forming tumors called “cerebral organoid glioma” (GLICO) (Figure 2B) [79]. The formed tumors resemble the features of the human disease; furthermore, these GSCs derived tumors exploit a network of microtubules to facilitate the multicellular connection between the tumor cells. Overall, we may assume that co-cultures of cerebral organoids represent an encouraging future opportunity to explore GB biology in a primitive human brain environment and to investigate the molecular mechanisms underlying tumor infiltration.

Two different studies tested 3D GB co-cultures for their response to various compounds, including temozolomide, the conventional drug used for GB treatment [98,99]. The results obtained underscore that the cell-cell contacts are crucial for the cooperation between different cell populations. This discovery has a relevant influence on the tumor response to drugs. In fact, comparing monocultures with co-cultures, it has been noticed a diminished sensitivity to treatments when more than one cell population is present in the culture, reinforcing the idea that not only tumor cells are involved in drug resistance (Table 2).

Overall, 3D co-culture glioblastoma tumor models hold great potential as a tool for improving in vitro cancer drug screening, since the increased complexity of the environment could enhance the effective responsiveness to a drug treatment resembling the in vivo effect [13,100]

**Table 2:** Advantages and disadvantages of glioblastoma organoids.

Model	Advantages	Limitations	Future Perspectives
Genetic engineered cerebral organoids (NeoCOR) [46,77]	Functional analysis of GB-related mutations Interaction between transformed and not transformed cells	Non representative of patients’ heterogeneity	Co-cultures with stroma and immune cells to assess TME interactions
Co-cultures with tumor spheroids (GLICO) [78,79]	Study patient-specific GBs High-throughput drug screening Partially recapitulates TME	Time consuming due to spheroids derivation	*In vivo* validation of in vitro drug screening results
Patients derived organoids [75,76]	Retain patient-specific heterogeneity Recapitulates tumor environment Fast organoid derivation (>2 weeks) GBOs Biobank	Prone to diverge from primary tumor over time	Improvement of immunotherapy approaches

## 5. Patient-Derived Glioblastoma Organoids

Every tumor exhibits a high grade of complexity and heterogeneity which develops during the neoplastic process [101]. Indeed, glioblastoma is an excellent example of this rule. However, this fact complicates the understanding of its biology and the prediction of patients overall survival [44]. In recent years, a significant effort has been made to establish patient-derived organoids that could retain the parental tumor heterogeneity, a relative 3D spatial organization and fundamental interactions with the ECM [102,103,104,105,106].

In 2016, for the first time, the laboratory of Jeremy Rich was capable of deriving glioblastoma organoids from finely minced tumor biopsies of both patients and genetically modified GB mice models [75]. These Matrigel^TM^ embedded 3D structures, compared to the tumor-spheres, presented several advantages such as the specimens’ size that could reach 3–4 mm in 2 months versus the typical 300 µm size of neurospheres (Figure 2C). On the other hand, GB organoids were stable for more than one year in culture and, once orthotopically implanted, initiated a highly diffuse and infiltrative glioma instead of expanding as solid sheets that are typical for glioblastoma xenografts [75]. Notably, the authors observed inside the organoids a hypoxic core characterized by a low amount of SOX2^+^ senescent stem cells, while the periphery presented a high density of highly proliferating SOX2^+^ stem cells, characterized by different molecular markers [75]. The inverse relationship between stem cell density and oxygen gradient is similar to what occurs in vivo, where limitations in oxygen and nutrients stimulate glioblastoma self-renewal and promote maintenance of a stem-like cell state [29,107]. In conclusion, GB organoids allowed the co-culture of phenotypically diverse stem and non-stem glioblastoma cells, opening new avenues for future studies on cancer development and tumor hierarchy. However, further validations of the model across several types of GB are still lacking and the long time required to establish the cultures (1–2 months) is not compatible with translational studies, since the aggressiveness of the pathology.

More recently, a faster protocol for the derivation of GB organoids (here called GBOs) was developed [76]. This system allowed to obtain GBOs in only one or two weeks starting from 1-mm biopsy dissections. The protocol has been optimized to better retain parental cytoarchitecture, heterogeneity and cell-cell interactions, thus preventing clonal selection of specific cell populations in culture. To address these challenges, the authors excluded mechanical and enzymatic dissociation of the collected tissue and used a fully defined serum free medium with no addiction of EGF/FGF2, two growth factors usually needed for neural and GB stem cells expansion [40,108]. Moreover, GBOs were cultured in an orbital shaker to facilitate the organoid formation and to guarantee homogeneous diffusion of nutrients and oxygen (Figure 2D). Comparably to the previously analyzed glioblastoma organoid model, GBOs presented a hypoxia gradient. Simultaneously, immunostaining analysis and RNA-seq revealed similarities in the heterogeneity of organoids respect to parental tumors (e.g., comparable percentage of SOX2^+^ and OLIG2^+^ cells), as well as cell proliferation rates. GBOs establishment is fast and reproducible, presenting high reliability with the parental tumor, making them suitable for drug testing in a scenario of personalized medicine approaches. Indeed, the authors have already successfully tested some specific drugs, such as EGFR or mTOR inhibitors, and CAR-T treatments on GBOs. These results highlight the potentialities of having a biobank of patient-derived glioblastoma organoids [109] which they established from 53 patient cases carrying a variety of genomic alterations commonly found in glioblastomas [76] (Table 2).

Although tumor organoids recapitulate some details of the TME better than tumor-induced organoids, such as gradients of stem cells and hypoxia, they still need in vivo studies to investigate their interactions with the healthy tissue. However, up to now, GBOs are probably the most suitable in vitro model for reproducing the patient heterogeneity and thus for exploring personalized therapeutic strategies [110] (Table 2).

## 6. Conclusions

Here, we describe novel 3D GB models with a particular focus on glioblastoma organoids, which have recently risen as encouraging platforms to investigate GB tumor biology. Traditional GB models fail to resemble tumor complexity. By contrast, 3D models better mirror patients’ heterogeneity, closely mimicking drug response observed in clinics and providing a robust and stable model for translational research.

Despite researchers’ attempts to build more refined GBM models, some relevant issues still need to be addressed. Up to date, none of these technologies is fully able to integrate human cancer heterogeneity with microenvironmental cues present both in tumor and surrounding healthy tissue. An attempt in this direction was carried out by Cui and colleagues, who generated a glioblastoma-on-a-chip model by bioprinting together patient-derived cancer cells and vascular endothelial cells [111]. The model accurately recapitulates some features of human pathology, such as intratumoral hypoxia gradient and an accurate TME. However, contrary to organoids, cells are not able to self-assemble, leading to an incorrect 3D structure. Moreover, bioprinting is still quite expensive and advanced expertise in performing the technique is required.

A more feasible strategy might be represented by performing co-cultures of early-stage organoids with patient-derived endothelial cells, which can be directly embedded in Matrigel, forming capillary-like structures around organoids [14,70]. This approach was shown to be promising to study environmental interactions with healthy brain organoids, however it still needs more validation. Moreover, to our knowledge, this co-culture method has not yet been tested to investigate the interaction between endothelial cells and tumoroids formation.

Further efforts should be also directed toward the dissection of the role of inflammation and immune cells in the crosstalk between GB cancer cells and the surrounding microenvironment [13]. At present, GBOs have been exploited to investigate immunotherapy approaches. Indeed, GBOs were shown to closely resemble the endogenous antigen expression, thus providing a reliable model to study the efficacy of CAR-T based treatments [76]. Again, it would be intriguing to study the relationship between GBM tumors and the immune system using co-culture models [14]. Tumoroid formation recapitulates the major steps involved in tumorigenesis, thus providing important insights on aberrant pathways. This acquired knowledge would favor the possibility to unveil new therapeutic targets that may dramatically reduce the incidence of recurrence rate and ameliorate prognosis.

Moreover, new rising technologies, such as 4D real imaging [112], microfluidics [113], organ-on-a-chip technology [111], and single cells sequencing [68], will surely be exploited to unveil novel insights on GB tumoroids biology, uncovering unexplored potentials of these models. Overall, GB organoids have already raised many hopes and it is reasonable that their potential will grow further in the near future, eventually leading to a personalized glioblastoma therapeutic approach.
